# Angular limb deformity associated with *TSPAN18*, *NRG3* and *NOVA2* in Rambouillet rams

**DOI:** 10.1038/s41598-023-43320-6

**Published:** 2023-09-25

**Authors:** Gabrielle M. Becker, Katie A. Shira, Julia L. Woods, Sarem F. Khilji, Christopher S. Schauer, Brett T. Webb, Whit C. Stewart, Brenda M. Murdoch

**Affiliations:** 1 Department of Animal, Veterinary and Food Science, University of Idaho, Moscow, ID USA; 2 Hettinger Research Extension Center, North Dakota State University, Hettinger, ND USA; 3 Department of Veterinary Sciences, University of Wyoming, Laramie, WY USA; 4 Department of Animal Science, University of Wyoming, Laramie, WY USA

**Keywords:** Genetics, Diseases

## Abstract

Angular limb deformity (ALD) affects many species of livestock and companion animals. The mechanisms of ALD development are not well understood, but previous research suggests the involvement of genetic risk factors. A case-control genome-wide association study (GWAS) was conducted with 40 ALD-affected and 302 unaffected Rambouillet rams and 40,945 single nucleotide polymorphisms (SNPs). Forelimbs of 6 ALD-affected rams were examined and diagnosed with osteochondrosis. Genome-wide or chromosome-wide significant SNPs were positioned exonic, intronic or within the 3′UTR of genes *TSPAN18*, *NRG3* and *NOVA2*, respectively. These genes have previously described roles related to angiogenesis and osteoblast, osteoclast and chondrocyte proliferation and differentiation, which suggests the possibility for their involvement in the pathogenesis of osteochondrosis. Functional consequences of SNPs were evaluated through transcription factor binding site analysis, which predicted binding sites for transcription factors of known importance to bone growth, including SOX6, SOX9 and RUNX2. The identification of genetic risk factors for ALD may help to improve animal welfare and production in Rambouillet, a breed known to be at risk for ALD development. This study proposes genes *TSPAN18*, *NRG3* and *NOVA2* as targets for further research towards understanding the etiology of ALD in Rambouillet sheep.

## Introduction

Angular limb deformity (ALD) is a welfare and longevity concern for livestock and domestic animals. The term ALD describes deviations in the frontal plane of the limb that can be medial or lateral, unilateral or bilateral and congenital or acquired^1^. To the best of our knowledge there is no single disease pathway responsible for ALD. Congenital ALD has been associated with perinatal factors such as maternal nutrition, teratogen exposure, premature birth, twin pregnancy or placentitis^1,2^. The causes of acquired ALD are likely multifactorial and may involve the interaction of trauma, genetics, rapid growth rate, endocrinological and/or nutritional factors^3,4^. Dietary mineral imbalance, such as of vitamin D, calcium, phosphorus, copper, zinc or magnesium, have also been known to contribute to ALD^3,5^. Evidence in multiple species strongly suggests a genetic component to ALD etiology, which may function in conjunction with other predisposing environmental influences^2,6,7^.

Osteochondrosis is frequently described as the underlying pathology of acquired ALD in sheep, cattle, horses, pigs, dog and poultry^8,9^. Osteochondrosis is a non-infectious disorder characterized by focal disturbance of endochondral ossification that may affect the articular cartilage (osteochondrosis dissecans) or the physes (physeal osteochondrosis) of the long bones of animals and humans^10^. These disturbances may be caused by hypertrophic chondrocytes that fail to progress or a lack of chondrocyte organization in the proliferative zone, which ultimately lead to areas of retained cartilage promoting lameness and leg weakness^11,12^. The development of osteochondrosis is strongly associated with high-energy diets and rapid growth, and it is thought to involve the interaction of genetic, nutritional and mechanical factors, with no one factor being entirely responsible for disease progression^9,12^. Previous studies have reported genetic regions associated with osteochondrosis in horses and pigs, although specific genetic mechanisms remain undetermined^13,14^.

Incidence of acquired ALD have been observed in Rambouillet rams during central performance ram testing at North Dakota State University (NDSU) and University of Wyoming (UWY). In the present study, a subset of affected rams were examined with gross and microscopic pathology to better understand the cause of ALD in these animals. The relatively low rates of disease development suggest the contribution of genetic risk factors; therefore, genotype data were collected in order to test for SNPs associated with ALD. Genome-wide association study (GWAS) was conducted with 40 ALD-affected and 302 unaffected Rambouillet rams who were enrolled in NDSU and UWY ram tests over a three-year period. Three different models were evaluated for comparison and interpretation of GWAS results, and the significant GWAS markers and corresponding genes were investigated for known biological roles related to bone growth and development.

## Results

### Central performance ram test

**Figure 1:**
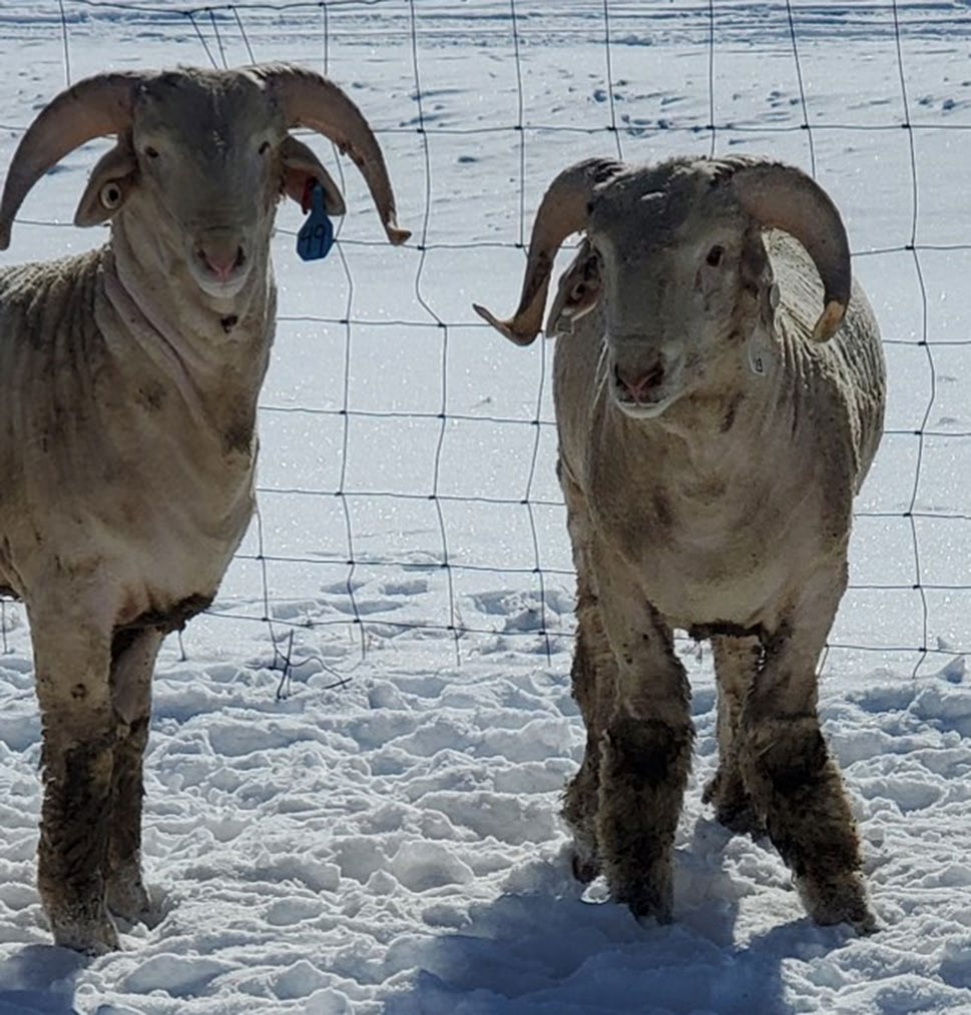
Visual presentation of ALD in a Rambouillet ram from UWY. The ram to the left demonstrates normal structure of the forelimbs and the ram to the right displays carpal valgus, indicative of ALD.

Rams were 7 ± 3 months of age at the time of enrollment in the NDSU and UWY central performance ram tests. Of the 342 Rambouillet rams collected from ram tests in 2019, 2020 and 2021, 40 rams developed ALD and 302 rams remained unaffected by conclusion of the 140-day testing period. Rams were considered to be affected by ALD upon presentation of abnormal conformation (valgus or varus deviations) of one or both forelimbs, as determined by trained UWY and NDSU sheep center staff who had daily interactions with the animals (Fig. 1). Disease incidence varied from 2.70 to 21.67% by year and location, with an average of 14.04% of NDSU and 9.83% of UWY rams developing ALD, and an overall study incidence rate of 11.70% (Supplementary Table S1). A consistent nutritional profile was provided in each year of the study.

### Gross pathology and histopathology examination

The examined forelimbs of six ALD-affected rams displayed varying degrees of carpal valgus with occasional slight deviation of the fetlock. The humerus, olecranon, radius, metacarpal and phalanges were sectioned and examined. Gross changes consisting of mild to moderate irregular physeal thickening primarily affected the distal radial physes, although some mild changes were observed in the olecranon physis of two rams and the distal metacarpal physis of one ram.

**Figure 2:**
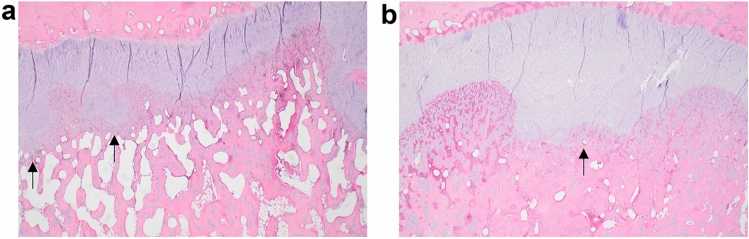
Frontal sections of distal radius from two ALD-affected animals. Images show mild (**a**) to moderate (**b**) irregular physeal thickening primarily affecting the distal radial physes (arrows). The irregular physeal thickening consists of both the proliferating and hypertrophic zones which extend down into the metaphysis.

The histologic changes were similar amongst the different animals and varied only in the degree of severity. Changes were primarily characterized by irregular thickening of the physis (growth plate) consisting of both the proliferating and hypertrophic zones that extend down into the metaphysis (Fig. 2). Occasional foci of retained cartilage were present in the metaphysis and epiphysis. Chondrocyte maturation appeared normal with trabecular cartilage cores lacking appreciable chondrocytes. Evidence of increased fragility such as microfractures were not observed. The histological changes of all ALD-affected forelimbs were consistent with physeal osteochondrosis which reflected an underlying failure of endochondral ossification.

### Genome-wide association study

**Figure 3:**
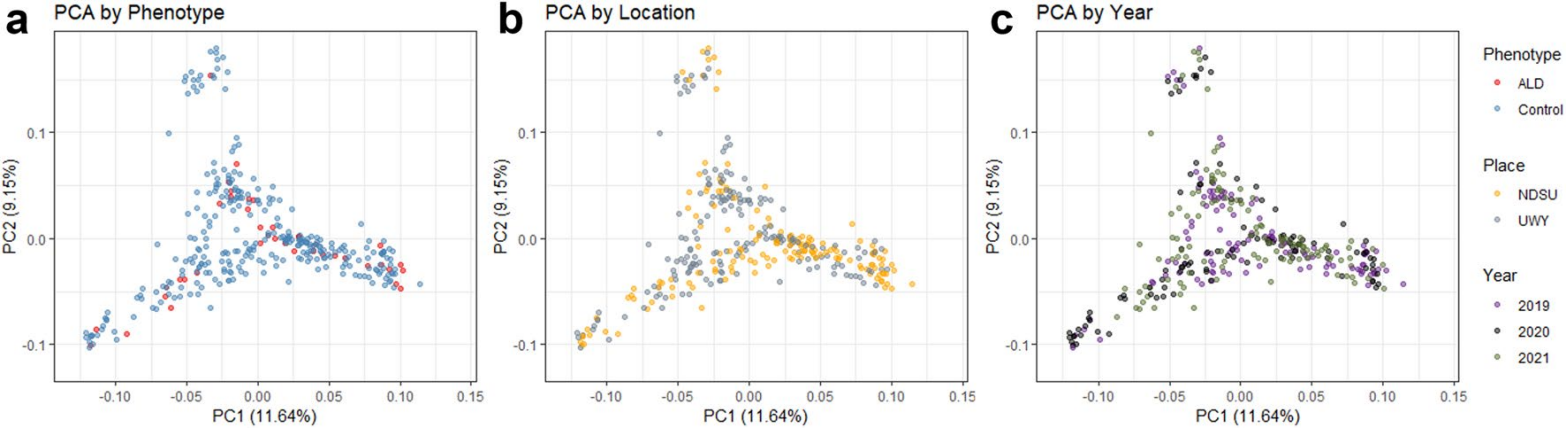
Principal component analysis (PCA) of Rambouillet rams. In each panel, the PC1 is plotted on the x-axis and PC2 is plotted on the y-axis. (**a**) PCA with rams coded by phenotype, given by: red, ALD-affected rams; light blue, control rams; (**b**) PCA with rams coded by testing location, given by: yellow, NDSU; gray, UWY; (**c**) PCA with rams coded by test year, given by: violet, rams from test year 2019; black, 2020; green, 2021.

Population structure and its relationship to phenotype, test location and test year were evaluated through principal component analysis (PCA). The first principal component (PC1) had an eigenvalue of 9.62 and explained 11.64% of the total genetic variance and the PC2 had an eigenvalue 7.56 and explained 9.15% of the total genetic variance. There did not appear to be any specific grouping of ALD-affected animals apart from control animals by either PC1 or PC2 (Fig. 3a). Similarly, rams from NDSU and UWY appeared to be evenly represented across PC1 and PC2 (Fig. 3b), as were rams from study years 2019, 2020, and 2021 (Fig. 3c).

**Figure 4:**
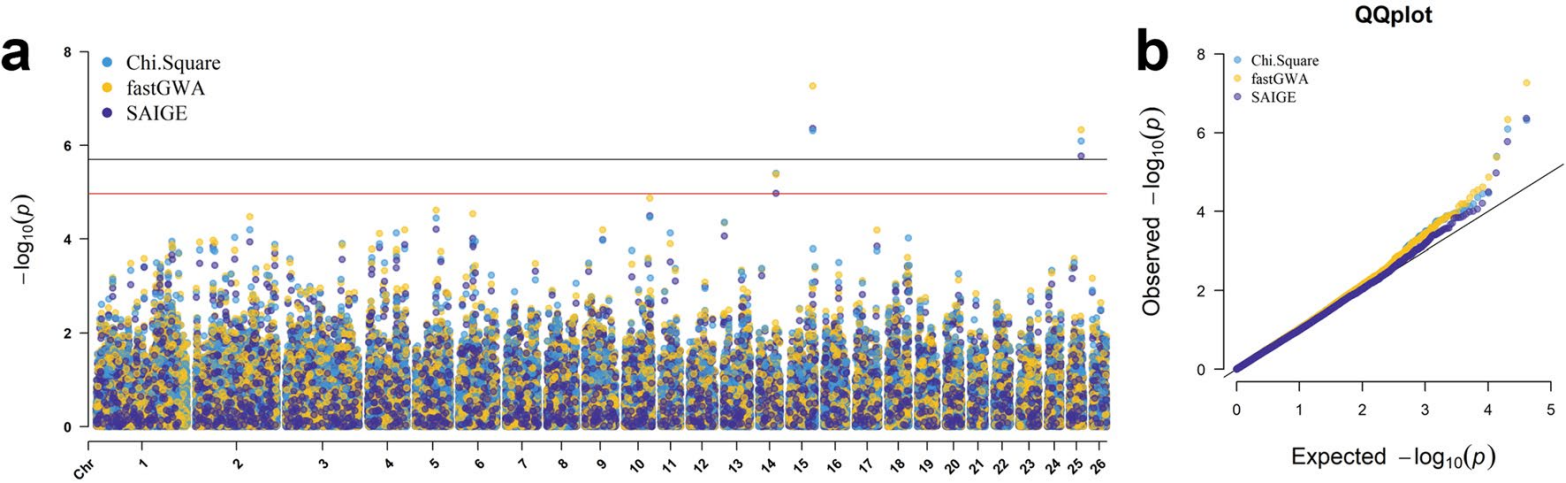
Multi-Manhattan and QQ plots displaying genome-wide association study results for incidence of ALD in Rambouillet rams. (**a**) Multi-Manhattan plot for the chi-square, fastGWA, and SAIGE models. Genome-wide significance is represented by the black line and defined by permutation testing (*P*-values < 2.00e−06); chromosome-wide significance is represented by the red line and defined by Bonferroni correction of the largest chromosome (*P*-values < 1.08e−05). (**b**) Quantile–quantile (QQ) plot for the chi-square, fastGWA, and SAIGE models displaying the expected versus observed *P*-values for each model.

Genome-wide association study models were investigated using fastGWA linear regression in GCTA software, the Scalable and Accurate Implementation of GEneralized mixed model (SAIGE) R package, and a 1 degree of freedom (df) chi-square allelic test in plink software. In all models, the same three SNPs were significant by Bonferroni chromosome-wide (*P*-values < 1.08e−05) and/or permutation genome-wide (*P*-values < 2.00e−06) significance thresholds: rs160736723 located on chromosome 15, rs427563170 on chromosome 25, and rs416810983 on chromosome 14 (Fig. 4a). The estimated odds ratio (OR) of significant SNPs were 4.77, 4.03 and 3.50, respectively, and all OR were supported by 95% confidence intervals (Table 1, Supplementary Fig. S1). The SNP minor allele frequencies (MAF) of ALD-affected rams were 20.00%, 25.00% and 27.50%, compared to 4.98%, 7.64% and 9.77% for unaffected rams (Table 1). The genomic inflation factor (λ_GC_) for the fastGWA, SAIGE, and chi-square models were 1.05, 1.02, and 1.06, respectively (Fig. 4b), indicating a lack of appreciable inflation of GWAS *P*-values^15^.

**Table 1:** Significant SNPs identified through three genome-wide association study models for ALD incidence.

Rs number	Variant type	Chr:position (bp)	ALD MAF (%)	Control MAF (%)	fastGWA *P*-value^+^	SAIGE *P*-value*	Chi-square *P*-value^	Chi-square (1 DF)	OR	95% CI
rs160736723	Synonymous (*TSPAN18*)	15:73,892,910	20.00	4.98	5.41e−08	4.36e−07	4.87e−07	25.32	4.77	2.47–9.22
rs427563170	Intronic (*NRG3*)	25:36,774,810	25.00	7.64	4.69e−07	1.68e−06	8.06e−07	24.34	4.03	2.24–7.26
rs416810983	Downstream (*NOVA2*)	14:53,230,561	27.50	9.77	4.22e−06	1.06e−05	3.98e−06	21.28	3.50	2.00–6.13

Marker positions are given for reference genome ARS-UI_Ramb_v2.0. The unadjusted *P*-values for the fastGWA, SAIGE, and chi-square models are reported.

*ALD* angular limb deformity, *CI* confidence interval, *DF* degree of freedom, *MAF* minor allele frequency, *OR* odds ratio.

^+^fastGWA run in GCTA version 1.93.2; *SAIGE run in SAIGE v0.45 R package; ^Chi-square model run in plink v1.9 software.

### Genomic context of significant SNPs

Genome-wide significant SNPs were located within genes tetraspanin 18 (*TSPAN18*; rs160736723, synonymous variant) and neuregulin 3 (*NRG3*; rs427563170, intronic variant). Additionally, the gene tumor protein p53 inducible protein 11 (*TP53I11*) and the uncharacterized ncRNA LOC121816715 were located within the region defined by ± 100 kb of SNP rs160736723. The chromosome-wide significant marker rs416810983 was located in a gene-rich region containing 10 genes within ± 100 kb (Supplementary Table S2) and was positioned 1,961 bp downstream in the unannotated 3′ UTR of the gene, NOVA alternative splicing regulator 2 (*NOVA2*). Although the 3′ UTR of *NOVA2* is annotated as 352 bp in length in the sheep reference genome (ARS-UI_Ramb_v2.0), the assemblies of human and mouse contain much longer annotations; the *Mus musculus NOVA2* 3′ UTR is 6,701 bp (GRCm39 C57BL/6J genome) and the *Homo sapiens NOVA2* 3′ UTR annotation is 6,129 bp (GRCh38.p14 genome). Reference RNA tracks imply that the *NOVA2* 3′ UTR is a similar length in sheep, as RNA signal extends from the gene to roughly 5552 bp downstream according to the “RNA-seq exon coverage” track in the NCBI Genome Data Viewer. Therefore, it is likely that SNP rs416810983 is located within the *Ovis aries NOVA2* 3′ UTR.

### Functional context of significant SNPs

The SNPs and corresponding genes implicated through GWAS were further investigated to better understand their potential functional context. Significant markers were located either exonic, intronic or in the 3′ UTR, suggesting the possibility for SNP alleles to be located within transcription factor binding site (TFBS) motifs^16^. The SNPs rs160736723, rs427563170 and rs416810983 were queried through the search tool FABIAN to compare against known TFBS motifs within the JASPAR 2022 database. Seventeen TFBS were predicted with score differences greater than ± 0.1 between reference and alternate SNP alleles, indicating either loss or gain of motif specificity (Table 2). The predicted TFBS included transcription factors with known relevance to bone growth, including RUNX2, NFE2L1, TBX5 at rs160736723, CREB1, CREM and SOX9 at rs427563170 and SOX6 at rs416810983.

**Table 2:** Results of TFBS predictions from reference genome sequence of significant SNPs.

Marker	Associated gene	Predicted TFBS	Score	Variant effect	Relevant citations
rs160736723	*TSPAN18*	TFAP2B	0.6051	Gain	Limb development^17^; candidate for growth traits in sheep^18^
		TFAP2A	0.4534	Gain	One of the Smad-partner TFs^19^; Smad contributes to osteogenic regulation^20^
		ARNT (HIF-1b)	0.1143	Gain	HIF promotes increased bone mass and angiogenesis^21^
		TBX5	− 0.1007	Loss	Candidate gene for osteochondrosis in pig^13^
		NFE2L1	− 0.1065	Loss	Regulator of osteoclastogenesis^22^
		RUNX2	− 0.1485	Loss	Major roles in chondrocyte hypertrophy and bone growth^23^
		MEIS1	− 0.7295	Loss	Importance in hematopoiesis^24^
rs427563170	*NRG3*	RORB	0.4995	Gain	Potential roles in chondrogenesis^25^
		CREB1	0.4976	Gain	CREB family involved in regulating chondrocyte proliferation^26^
		CREM	0.2684	Gain	CREM family may regulate TFs involved in osteoclastogenesis and bone homeostasis^27^
		SOX9	0.2021	Gain	Essentail roles in chondrogenesis^28^
		NKX3-1	0.1551	Gain	Expressed in mesenchymal chondrosarcoma^29^
		CRX	0.1074	Gain	
rs416810983	*NOVA2*	NKX2-5	0.2233	Gain	
		CRX	0.1388	Gain	
		SOX6	− 0.1116	Loss	Regulation of chondrogenesis^28^
		E2F6	− 0.2566	Loss	Regulation of *Hox* genes in axial skeletal development^30^

Results with a score ± 0.1 are reported. The variant effect indicates whether the SNP alternate allele results in a gain or loss of TFBS compared to the SNP reference allele.

The STRING database was used to identify known and predicted protein-protein interactions of the genes implicated by GWAS results. The *Homo sapiens* orthologs of *TSPAN18*, *NRG3* and *NOVA2* were queried and results revealed five protein interactions with TSPAN18 and eight protein interactions each with NRG3 and NOVA2 (Table 3). Interactant proteins with notable biological roles relevant to bone health included ADAM10 with TSAPN18 and HNRNPK and MBNL1 with NOVA2. For NRG3, notable interactions included EGFR, ERBB3, ERBB4, HRAS, KRAS and HSP90AA1.

**Table 3:** Results of STRING database query for genes implicated by GWAS.

Protein of interest	STRING interactant	Coexpression score	Interaction score	Relevant citations
TSPAN18	ADAM10	–	0.105	Indirect regulation of hypertrophic chondrocytes and longitudinal bone growth; essential for processing Notch^31^
	CACNA1B	–	0.056	
	IGSF8	–	0.057	
	ZKSCAN4	–	0.056	
	ZSCAN31	–	0.056	
NRG3	EGFR	–	0.162	Critical for osteoprogenitor cell maintenance and bone formation^32^
	ERBB3	–	0.162	Promotes angiogenesis^33^
	ERBB4	0.139	0.312	Enhances angiogenesis through PI3K/AKT, MAPK/ERK pathways^34^
	HRAS	–	0.142	Responsible for VEGF induction; involved in neovascularization and vascular permeability^25,34^
	HSP90AA1	–	0.156	HSP90AA1 expression correlated with VEGF/VEGFR2-PI3K-AKT pathway activation^37^
	KRAS	–	0.142	KRAS pathway involved in upregulation of VEGF^38^
	NRG1	–	0.126	
	NRG2	0.076	0.15	
NOVA2	HNRNPK	0.062	0.612	Roles in osteogenesis; controls transcription of osteocalcin^39^
	MBNL1	0.064	–	MBNL1-AS1 highly expressed in blood vessels^40^
	PTBP2	0.049	0.182	
	RBFOX1	0.18	–	
	RBFOX2	0.094	–	
	RBFOX3	0.157	–	
	SCN1A	0.105	–	
	SRRM4	0.149	–	

The table describes known protein association networks for genes *TSPAN18*, *NRG3* and *NOVA2.*

## Discussion

Osteochondrosis has previously been described as a cause of ALD in sheep, horses, pigs and other species^8,9^. Physeal osteochondrosis is characterized by lesions of retained cartilage in the bone resulting from the focal failure of endochondral ossification. Disease development is thought to require several contributing factors, including high-energy diet, rapid growth rate and genetics^12^, and early lesions may heal and never progress to clinical disease^41^. Although a number of biological mechanisms have been proposed, failure of blood delivery to the growth cartilage is thought to be the initiating event in disease pathogenesis^8^. Chondrocyte-driven lengthening of long bones is an intricate process that involves the coordinated control of transcription factors, growth factors and cytokines, that culminates in endochondral ossification, the replacement of avascular cartilage with highly vascularized bone^42^. Immature chondrocytes direct chondrocyte differentiation through expression of SOX5, SOX6 and SOX9 transcription factors. These cells also produce the extracellular matrix components collagen type II α1 and aggrecan^43^. Hypertrophy is initiated by downregulation of SOX5, SOX6, SOX9 and collagen type II α1, accompanied by the simultaneous upregulation of RUNX2, which regulates both chondrocyte maturation and osteoblast differentiation^12,42^. Hypertrophic chondrocytes produce type X α1 collagen to create the matrix template for later calcification^42–44^. In later stage hypertrophy, chondrocytes produce vascular endothelial growth factor (VEGF), matrix metalloproteinase 13 (MMP13) and osteopontin to direct angiogenesis and the invasion of blood cells into the cartilage matrix. These vessels facilitate the delivery of osteoclasts, osteoblasts, and bone marrow cells to the ossification front, which orchestrate cartilage resorption and the deposition of mineralized bone^42,43,45,46^. Vascular invasion of the cartilage-bone interface is critical for matrix calcification and successful endochondral ossification^12^.

**Figure 5:**
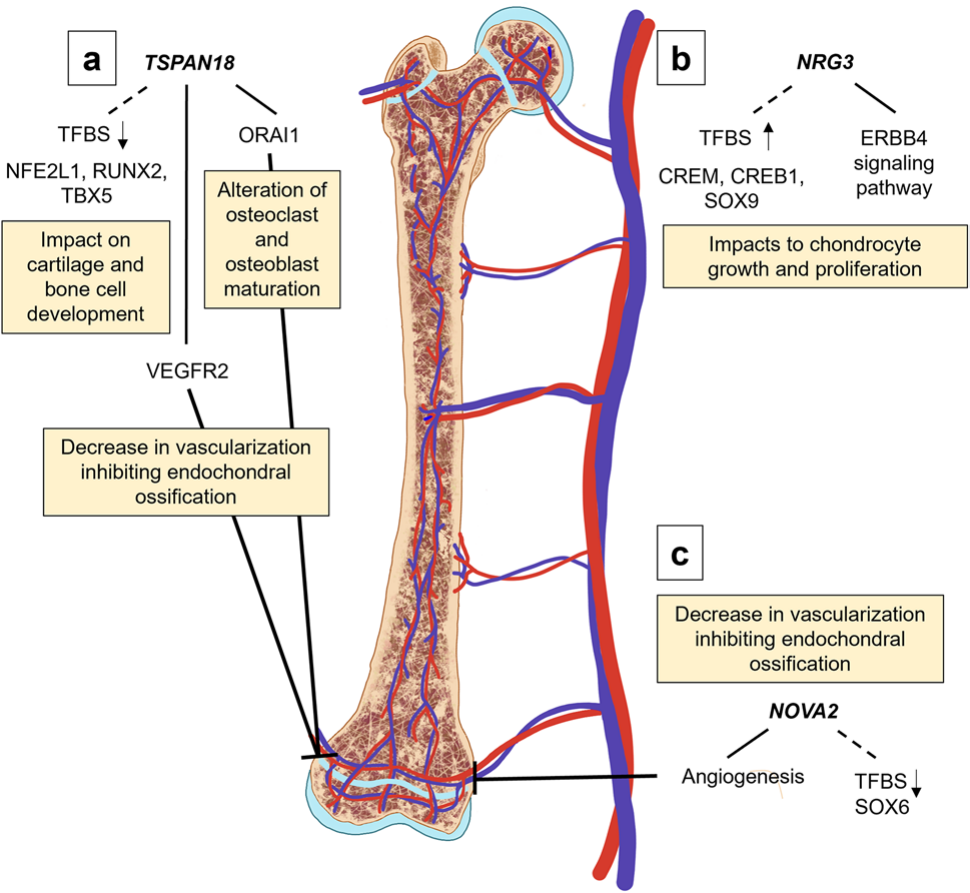
Summary of biological implications of GWAS results. (**a**) Deficiency of TSPAN18 gene expression may have negative repercussions on endochondral ossification through the downregulation of VEGFR2 leading to inhibition of the VEGF pathway and angiogenesis, or through decreased cell-surface expression of ORAI1 resulting in reduced Ca^2+^ signaling affecting osteoclast and osteoblast cell maturation. Predicted transcription factor binding site (TFBS) motifs, denoted by the dashed line, for NFE2L1, RUNX2 and TBX5 show loss of specificity with the rs160736723 SNP allele, potentially affecting TSPAN18 transcription; (**b**) Changes to NRG3 gene expression may impact chondrocyte growth and proliferation through the NRG3/ERBB4 pathway. Binding site motif specificities for transcription factors CREM, CREB1 and SOX9 are predicted to increase with the SNP allele at rs427563170; (**c**) Decreased gene expression of NOVA2 may decrease vascularization necessary for endochondral ossification. The SNP allele at rs416810983 results in decreased TFBS motif specificity of SOX6.

The biological relevance of the results of this study are supported by the current literature. The genes and transcription factors implicated by this GWAS have previously described roles in angiogenesis and the differentiation and proliferation of cartilage and bone cells, indicating pertinence to endochondral ossification and suggesting potential roles in osteochondrosis development. The most significant GWAS result is SNP rs160736723, a synonymous variant within *TSPAN18* (Fig. 5a)*.* The gene *TSPAN18* has been found to be highly expressed in endothelial cells and promotes the cell surface expression of the store-operated Ca^2+^ entry (SOCE) channel pore-subunit ORAI1^47,48^. Experiments in mice have suggested that Orai1 has a critical role in bone homeostasis through the regulation of osteoblasts and osteoclasts^47^. The lack of SOCE in MC3T3-E1 pre-osteoblastic cells had an inhibitive effect on mineralized nodule formation and Orai1−/− stromal cells showed impaired mineral deposition in in vitro experiments. Similarly, osteoclastogenesis was impaired in Orai1−/− due to defects in the fusion of pre-osteoclasts^47^. In mouse and zebrafish, Tspan18 expression was noted in the vasculature of developing embryos and in the adult during wound healing. Deficiency of Tspan18 was found to result in the downregulation of VEGFR2, suggesting Tspan18 as either a direct or indirect regulator of the VEGF pathway^49^. Additionally, TSPAN18 has an experimentally determined interaction with ADAM10 (Table 3), a protease found to affect the long bone longitudinal growth in mice through the CXCL12/CXCR4 signaling axis^31^.

The significant SNP rs427563170 is an intronic variant within gene *NRG3* (Fig. 5b). The extracellular epidermal growth factor (EGF)-like domain of NRG3 is a ligand that binds to the extracellular domain of ERBB4 (HER4), a member of ERBB family of receptor tyrosine kinases^50^. ErbB4 has been shown to be expressed in developing mouse cartilage and bone cells^51^ and may have roles in chondrocytic growth and differentiation^52^. The STRING database indicates that NRG interacts with EGFR, a receptor protein critical for bone formation (Table 3), as well as with proteins ERBB3, HRAS, HSP90AA1 and KRAS that promote angiogenesis and neovascularization^32,34,36^.

The chromosome-wide significant SNP, rs416810983, is positioned within the 3′ UTR of *NOVA2* (Fig. 5c). NOVA2 is an alternative splicing regulator that is expressed in vascular endothelial cells and is essential for angiogenesis and vascular morphogenesis^53^. There are known STRING interactions and/or protein coexpressions of NOVA2 with MBNL1 and hnRNPK (Table 3). Expression of the lncRNA MBNL1 has been found to be upregulated in vascular smooth muscle cells during differentiation^40^. Transcription of osteocalcin, a factor that stimulates matrix mineralization, is repressed by hnRNPK. Additionally, hnRNPK has regulatory roles in osteogenesis through interactions with lncRNA‐OG in mesenchymal stem cells^39^.

The expression of *TSPAN18*, *NRG3*, and *NOVA2* may be regulated in part by transcription factors with known importance to bone growth. The alternate SNP allele at rs160736723 within *TSPAN18* was predicted to result in a loss of TFBS motif specificity for RUNX2, NFE2L1 and TBX5 transcription factors (Table 2). Runx2 is a major transcription factor in bone growth with regulatory roles in osteogenesis, chondrogenesis, chondrocyte maturation and osteoblast differentiation^23^. Importantly, chondrocyte hypertrophy, which initiates endochondral ossification, is controlled by upregulation of RUNX2^46^. Depletion of NFE2L1 (also known as NRF1) has been shown to impair osteoblast differentiation and osteoclastogenesis^22^. The gene *TBX5* was identified as a candidate for osteochondrosis development in pigs through GWAS and differential transcript abundance of healthy versus defect cartilage^13^. Haploinsufficiency of TBX5 is known to cause Holt-Oram syndrome in human patients, which is characterized in part by skeletal malformations^54^.

Predicted TFBS for SOX9, CREB1 and CREM showed gain of specificity with the alternate SNP allele at rs427563170 within *NRG3* (Table 2). Expression of SOX9 must be timed appropriately, as it is essential for chondrogenesis but prevents cartilage vascularization and successful endochondral ossification if not downregulated in the hypertrophic zone of the growth plate^28^. The CREB family are involved in regulating chondrocyte proliferation important for endochondral ossification^26^ and a member of the CREM family, ICER, is thought to regulate the activity and/or the expression of ATF/CREB transcription factors involved in osteoclastogenesis and bone homeostasis^27^. The predicted SOX6 TFBS showed loss of specificity with the rs416810983 alternate allele within the *NOVA2* 3′ UTR (Table 2). SOX6 is a member of the ‘SOX-Trio’ of transcription factors, together with SOX5 and SOX9, that regulate chondrogenesis^28^.

Many previous studies support a multifactorial etiology of osteochondrosis^4,10^, and a high plane of nutrition leading to rapid growth is often cited as a contributing factor of disease^12^. The high-energy diets fed during central performance ram tests are intended to provide balanced nutrition and support uninhibited growth to facilitate performance comparisons between animals^55^. We hypothesize that a combination of rapid growth and the presence of genetic risk factors likely lead to the development of clinical disease in the ALD-affected rams of this study. In view of the multifactorial nature of osteochondrosis, it is not surprising that the SNPs described in this study do not explain all of the disease variation. Unaffected rams with variant alleles may have had some level of subclinical osteochondrosis that was not observable or that resolved before progressing to clinical disease^41^ or may have not experienced enough environmental stress to facilitate lesion development.

This study suggests that the genes *TSPAN18*, *NRG3* and *NOVA2* may have biological functions in ALD disease progression. Despite the limited sample size, this study benefited from the environmental uniformity experienced by all affected and unaffected rams. Additionally, significant SNPs were identified through three different GWAS models, which lends confidence to these associations. Further research with a larger sample size is necessary to validate this study as well as to continue to elucidate the complex biological mechanisms that likely underpin ALD development.

In this study, Rambouillet rams with acquired ALD had gross and microscopic lesions consistent with physeal osteochondrosis. Rams with one or more alternate alleles at rs160736723, rs427563170 or rs416810983 were found to be 3.5 to 4.8 times more likely to develop ALD than rams with reference alleles. The genes implicated by GWAS results have previously described roles in angiogenesis, osteogenic differentiation and chondrocyte development and proliferation, suggesting their relevance to osteochondrosis. Further, the TFBS with predicted loss or gain of motif specificity at significant SNPs have known roles in the regulation and timing of endochondral ossification. Further work is necessary to validate GWAS findings and to elucidate the potential roles of *TSPAN18*, *NRG3* and *NOVA2* in the pathogenesis of physeal osteochondrosis. This study proposes three SNPs as genetic risk factors for ALD in Rambouillet rams and indicates potential genetic mechanisms that may contribute to disease.

## Methods

### Central performance ram test

Central performance ram testing was conducted as previously described (Becker et al. 2023). In each study year, ram lambs of 7 ± 3 months of age from more than 30 regional (WY, ND, SD, MT, CO) seedstock producers were brought to the University of Wyoming-Laramie Research and Extension Center (Laramie, WY; 41° 17′ N, − 105°40′ W) or North Dakota State University-Hettinger Research and Extension Center (Hettinger, ND; 46° 01′ N, − 102°65′ W). Rams were managed as a single cohort in a dry-lot management system at each testing location and were provided fully balanced ad libitum textured diets that met requirements for all phases of ram growth, containing 15% to 17% crude protein and 68% to 76% total digestible nutrients on a dry matter basis. Each ram test began in the fall and concluded in the spring and lasted for a total duration of 140 days. Ram test protocols were approved by North Dakota State University Institute for Animal Care and Use Committee (IACUC). All methods were carried out in accordance with relevant IACUC guidelines and regulations. The methods of this study have been reported in accordance with the ARRIVE guidelines.

### Gross pathology and histopathology examination

Nine forelimbs from six ALD-affected rams (three rams each from NDSU and UWY) were evaluated for gross and histological pathology. Each limb was evaluated grossly and the humerus, olecranon, radius, metacarpal and phalanges were sectioned. Decalcified, 5 µm thick, hematoxylin and eosin stained frontal section of the distal radius and the sagittal section of the olecranon of each submitted forelimb and the frontal section of the distal metacarpus of two forelimbs were evaluated. Images were taken on a BX 43 microscope, 2 × 0.08 NA plan apo lens on a DP 23 camera at 3088 × 2076 resolution.

### DNA genotyping and quality control

Whole blood or tissue samples were collected from 342 Rambouillet rams over a three-year period. Rams were sampled from NDSU in 2019 (n = 44), 2020 (n = 60), 2021 (n = 55), and from UWY in 2019 (n = 74), 2020 (n = 43) and 2021 (n = 66). The genotype data of 313 of these rams have been analyzed in previously published work^56^. DNA was isolated from blood at the University of Idaho using the phenol–chloroform method^57^ and tissue samples were provided to AgResearch for DNA extraction. The DNA samples were genotyped with the Applied Biosystems™ Axiom™ Ovine Genotyping Array (50K) consisting of 51,572 single nucleotide polymorphism (SNP) markers (Thermo Fisher Scientific, catalog number 550898) or the AgResearch Sheep Genomics 60K SNP chip consisting of 68,848 SNP markers (GenomNZ, AgResearch, New Zealand). Duplicate markers within a panel were filtered to retain the marker with the highest call rate (CR). Compatible markers between panels were matched by marker name and genome position and plink v1.9 was used to merge genotype array data^58^ (http://pngu.mgh.harvard.edu/purcell/plink/). Markers were filtered with plink v1.9 to remove non-autosomal markers and those with CR < 0.90, minor allele frequency < 0.01, and Hardy-Weinberg Equilibrium *P*-value < 1e−06. A total of 40,945 SNP markers were carried through to final analysis. All rams had a CR of 95% or greater.

### Genome-wide association study

Principal component analysis (PCA) was conducted to visualize the population structure and phenotypic distribution among ALD-affected and unaffected rams. Analysis was conducted with plink v1.9 and visualized with the packages ggplot2 and patchwork in R version 4.2.2^58–61^. Each plot was constructed with the first principal component (PC1) on the x-axis and the second principal component (PC2) on the y-axis. Rams were color-coded by phenotype (ALD-affected or unaffected), test location (NDSU or UWY) and test year (2019, 2020 or 2021). The proportion of variance explained by each PC were calculated by dividing the PC eigenvalue by the sum of eigenvalues.

In total 342 Rambouillet rams, including 40 ALD-affected and 302 unaffected, were examined in three genome-wide association study (GWAS) models. The case-to-control ratio of the study was 1-to-7.55, which approached but did not exceed the threshold of 1-to-10 generally used to define an imbalanced GWAS^62^. For this reason, three models with the ability to handle imbalance between study groups were chosen for analysis and comparison. The data were analyzed through a generalized linear mixed model (GLMM)-based fastGWA-GLMM in GCTA version 1.93.2 software^63^, a Scalable and Accurate Implementation of GEneralized (SAIGE) mixed model v0.45 available as a R package^62^, and a 1 degree of freedom (df) chi-square allelic test in plink v1.9 software^58,64^. In the fastGWA-GLMM, the genetic variance statistic (Vg) was not significant, likely due to the lack of highly related individuals within the dataset. Therefore, the program implemented the linear regression (fastGWA) model instead of GLMM^65^. The fastGWA model used a sparse genomic relationship matrix (GRM) to account for population stratification and was run with default parameters. The SAIGE model was run using the R scripts “step1_fitNULLGLMM.R” and “step2_SPAtests.R” with default minor allele count and frequency thresholds, and the leave one chromosome out (LOCO) method was used to construct a different GRM for each chromosome. In plink v1.9, the flags ‘–assoc’, ‘–adjust’, and ‘–ci 0.95’ were used to run the chi-square test and to report 95% confidence intervals for estimated odds ratio (OR) statistics. The GWAS results were visualized with the package CMplot and OR confidence intervals were visualized with gridextra and ggplot2 in R^60,66,67^.

To avoid over interpretation of results and to limit the possibility of Type I or Type II error, the genome-wide significance threshold for this study was determined by adaptive permutation^68^. Permutation testing was carried out in plink v1.9 (flags ‘–aperm’, ‘–assoc perm’). Each SNP underwent a minimum of 500 permutations and up to 1,000,000 permutations under default conditions for the pruning alpha threshold, pruning beta, initial pruning interval and the interval increase rate. The most significant *P*-value achieved by chance after 1,000,000 permutations was used to define the threshold for genome-wide significance. Chromosome-wide significance was calculated using the Bonferroni adjustment for the largest chromosome (chromosome 1; 4622 markers).

### Genomic context of significant SNPs

The database Ensembl^69^ was used to identify the variant type of significant SNPs. In order to understand the genomic contexts of SNPs, the reference genome ARS-UI_Ramb_v2.0^70^ was searched using the GenomeBrowser tool in NCBI^71^. Genes or noncoding elements located within ± 100 kb of significant SNPs were recorded for further investigation.

### Functional context of significant SNPs

Markers located within a gene or within the 3′ UTR were evaluated for predicted transcription factor binding site (TFBS) matrix score differences between reference and alternate SNP alleles. Reference genome sequences comprised of 11 bp total, with the SNP allele (reference or alternate) in the 6^th^ position, were queried through the online software FABIAN^72^. Sequences were tested against detailed transcription factor flexible models (TFFM) compiled within the JASPAR 2022 database^73^. The proteins of implicated genes were queried through the STRING database (using *Homo sapiens* orthologs) for known and predicted protein-protein interactions^74^. Interacting proteins and predicted transcription factors were investigated through literature review for potential relevance to bone health and growth.

### Approval for animal experiments

All ram test protocols were approved by North Dakota State University Institute for Animal Care and Use Committee (# 20210012). Informed consent was collected from all animal owners upon enrollment of their rams in the central performance ram test.

### Supplementary Information

Supplementary Information.

## Data Availability

All genotype data analyzed in this study have been made available from the EVA repository. Data accession details are as follows: project PRJEB58836, analyses ERZ15609617, available at https://www.ebi.ac.uk/eva/?eva-study=PRJEB58836.
