# Supplementary material for: Angular limb deformity associated with TSPAN18, NRG3 and NOVA2 in Rambouillet rams
Source: Sci Rep. 2023 Sep 25;13:16059. doi: 10.1038/s41598-023-43320-6 (PMC10520043; doi:10.1038/s41598-023-43320-6)
Supplement: Supplementary file 1 — Supplementary Information. [file 41598_2023_43320_MOESM1_ESM.docx]

**Supplemental Materials**

**Supplemental Tables**

Supplemental Table 1. Incidence rates of ALD by year and by central performance ram test location. NDSU, North Dakota State University; UWY, University of Wyoming.

| Test Year | Number of Rams | | Number of ALD Cases | | ALD Incidence (%) | | |
| --- | --- | --- | --- | --- | --- | --- | --- |
|  | NDSU | UWY | NDSU | UWY | NDSU | UWY | Combined |
| Total | 159 | 183 | 23 | 17 | 14.47 | 9.29 | 11.70 |
| 2019 | 44 | 74 | 5 | 2 | 11.36 | 2.70 | 5.93 |
| 2020 | 60 | 43 | 13 | 5 | 21.67 | 11.63 | 17.48 |
| 2021 | 55 | 66 | 5 | 10 | 9.09 | 15.15 | 12.40 |
| AVG | 53 | 61 | 7.67 | 5.67 | 14.04 | 9.83 | 11.94 |

Supplementary Table 2. Genes located within ± 100 kb of rs416810983. *The marker rs416810983 is a downstream (3’ UTR) variant of the gene *NOVA2*.

| **Gene symbol** | **Name** |
| --- | --- |
| *RSPH6A* | radial spoke head 6 homolog A |
| *SYMPK* | symplekin scaffold protein |
| *FOXA3* | forkhead box A3 |
| *IRF2BP1* | interferon regulatory factor 2 binding protein 1 |
| *MYPOP* | Myb related transcription factor, partner of profilin |
| *NANOS2* | nanos C2HC-type zinc finger 2 |
| *NOVA2** | NOVA alternative splicing regulator 2 |
| *CCDC61* | coiled-coil domain containing 61 |
| *PGLYRP1* | peptidoglycan recognition protein 1 |
| *LOC101120688* | insulin growth factor-like family member 1 |

**Supplemental Figures**


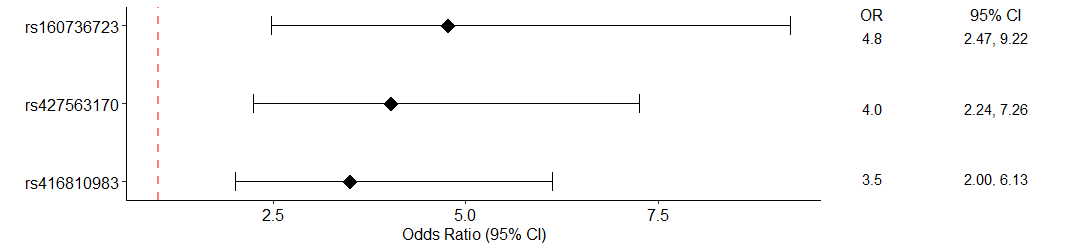


Supplemental Figure 1. Odds ratios and 95% confidence intervals for significant SNPs. Odds ratios indicate that rams with one or more variant alleles at significant SNPs are 3.5 to 4.8 times more likely to develop ALD than rams with only reference alleles.
